# ID 122 - Eficácia e Segurança do Dupilumabe em comparação ao Omalizumabe para Asma Grave com Fenótipo Alérgico: revisão sistemática com metanálises de comparações indiretas

**DOI:** 10.5327/2237-9622.2025.v34s1.122

**Published:** 2025-11-25

**Authors:** Aline Garcia Barbosa, Frederico Jose Bighetti Magro, Greice Ballardin Miotto Buttelli, Alexandre Taminato, Renata Prioli, Sarah Franco Watanabe, Bianca Salvador, Bruna Marmett, Celina Borges Migliavaca, Gilson Dornelles, Nayê Balzan Schneider, Maicon Falavigna

**Keywords:** asma, imunoterapia, revisão sistemática, metanálise em rede

## Abstract

**Introdução:**

A asma é caracterizada por inflamação crônica das vias aéreas. Entre seus fenótipos e endotipos, a inflamação tipo 2 (fenótipos alérgico e eosinofílico) é caracterizada por manifestação precoce e mais grave. Atualmente, o omalizumabe (OMA) é único imunobiológico disponível no SUS para o tratamento da asma grave com fenótipo alérgico. Recentemente, o dupilumabe (DUPI) – um anticorpo monoclonal humano antagonista do receptor da IL-4 e da IL-13 – surgiu como alternativa para essa condição.

**Método:**

Foram conduzidas duas revisões sistemáticas avaliando a efetividade do DUPI e do OMA em relação ao placebo no tratamento da asma grave com fenótipo alérgico, definido como IgE ≥30 UI/mL e sensibilidade a um ou mais aeroalérgenos perenes. Apenas estudos clínicos randomizados, com mascaramento de participantes e avaliadores, foram incluídos, devido ao componente subjetivo na definição de exacerbação. Também foram incluídos apenas estudos que avaliavam a adição de OMA ou DUPI ao tratamento com beta-agonistas de ação prolongada e corticosteroides inalatórios, conforme PCDT de asma. As buscas foram realizadas nas bases MEDLINE (PubMed), Embase e Cochrane CENTRAL, em janeiro de 2024. O risco de viés dos estudos individuais foi avaliado com o instrumento Risk of Bias 2.0, com a certeza da evidência sendo avaliada com o sistema GRADE. A partir dos resultados identificados nas duas revisões independentes, foi realizada metánalise de comparações indiretas. As análises foram feitas com o software R, pacotes meta e netmeta, utilizando abordagem frequentista.

**Resultados:**

Quatro ensaios clínicos avaliaram DUPI e oito avaliaram OMA. Em comparação ao placebo, DUPI reduziu o risco de exacerbações em 52% (RR 0,48; IC 95% 0,37 a 0,62; alta certeza da evidência) e aumentou o VEF1 em 0,16 L (IC 95% 0,12 a 0,20L; alta certeza da evidência), além de melhorar a qualidade de vida e o controle da doença. OMA, em comparação ao placebo, reduziu as exacerbações em 26% (RR 0,74; IC 95% 0,64 a 0,86; baixa certeza da evidência por risco de viés e evidência indireta) e aumentou o VEF1 em 0,08 L (IC 95% 0,04 a 0,11 L; baixa certeza por risco de viés e evidência indireta). Comparação indireta mostrou que DUPI reduz a taxa de exacerbações em 32% em relação ao OMA (RR 0,68; IC 95% 0,54 a 0,88; baixa certeza da evidência por risco de viés e evidência indireta em OMA) e aumenta o VEF1 em 0,08L (IC 95% 0,03 a 0,13; p = 0,001; baixa certeza por risco de viés e evidência indireta em OMA). No desfecho de descontinuação por eventos adversos, não houve diferença estatística entre os tratamentos, embora tenha sido observada uma redução de 26% com o DUPI comparado ao OMA (RR 0,74; IC 95% 0,28 a 1,93; moderada certeza da evidência por imprecisão).

**Conclusão:**

OMA e DUPI são eficazes no tratamento da asma grave com fenótipo alérgico, com a incorporação do OMA pelo SUS em 2019 sendo um marco importante para a atenção ao paciente com asma grave no SUS. A comparação indireta indica que DUPI está provavelmente associado à menor taxa de exacerbações e à melhora da função pulmonar em relação ao OMA, tornando-se uma opção viável para inclusão no SUS. Apesar dos resultados promissores, é essencial contextualizar o impacto clínico com aspectos econômicos para garantir a sustentabilidade do sistema com a adoção de tratamentos custo-efetivos.

